# Spin relaxation in antiferromagnetic Fe–Fe dimers slowed down by anisotropic Dy^III^ ions

**DOI:** 10.3762/bjnano.4.92

**Published:** 2013-11-27

**Authors:** Valeriu Mereacre, Frederik Klöwer, Yanhua Lan, Rodolphe Clérac, Juliusz A Wolny, Volker Schünemann, Christopher E Anson, Annie K Powell

**Affiliations:** 1 Institute of Inorganic Chemistry, Karlsruhe Institute of Technology, Engesserstr. 15, D-76128 Karlsruhe, Germany; 2 CNRS, CRPP, UPR 8641, F-33600 Pessac, France; 3 Univ Bordeaux, CRPP, UPR 8641, F-33600 Pessac, France; 4 Institute of Physics, University of Kaiserslautern, Erwin Schrödingerstr. 56, D-67653 Kaiserslautern, Germany; 5 Institute of Nanotechnology, Karlsruhe Institute of Technology, Postfach 3640, D-76021 Karlsruhe, Germany

**Keywords:** anisotropy, dysprosium, iron, Mössbauer spectroscopy

## Abstract

By using Mössbauer spectroscopy in combination with susceptibility measurements it was possible to identify the supertransferred hyperfine field through the oxygen bridges between Dy^III^ and Fe^III^ in a {Fe_4_Dy_2_} coordination cluster. The presence of the dysprosium ions provides enough magnetic anisotropy to “block” the hyperfine field that is experienced by the iron nuclei. This has resulted in magnetic spectra with internal hyperfine fields of the iron nuclei of about 23 T. The set of data permitted us to conclude that the direction of the anisotropy in lanthanide nanosize molecular clusters is associated with the single ion and crystal field contributions and ^57^Fe Mössbauer spectroscopy may be informative with regard to the the anisotropy not only of the studied isotope, but also of elements interacting with this isotope.

## Introduction

The huge Ising-type magnetic anisotropy of many lanthanide ions, which can be controlled by designing the ligand field, can slow down the relaxation of magnetisation and can be an effective source for the modulation of properties of transition metal molecular magnets [1]. The anisotropy of the lanthanide is severely affected by the symmetry of the crystal field and it can be controlled by a suitable design of the ligand field environment [2–3]. Today, the orientation of the principal axes of the magnetization of the lanthanide ions in low-symmetry environments can be determined theoretically and experimentally [4–9]. For molecular magnetism the Dy^III^ ion has proved to be the most attractive [4–12] not only because of its large flexibility regarding the interaction between the single-ion electron density and the crystal field environment, and its predicted hard anisotropy using simple ligand field considerations, but also because of its huge field dependence of the relaxation time [13].

**Figure 1:**
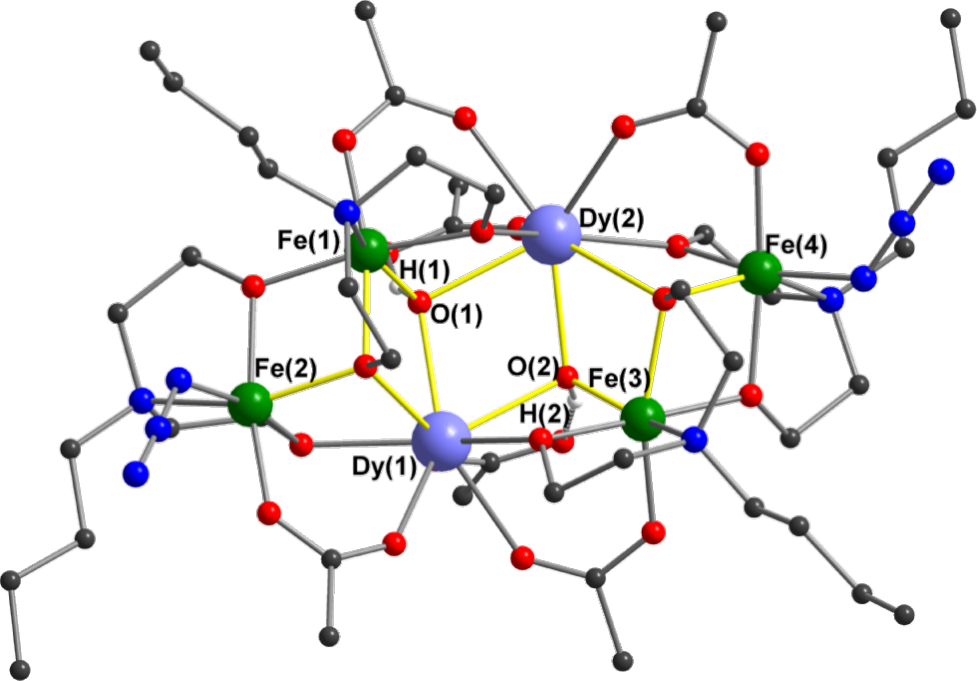
Molecular structure of [Fe_4_Dy_2_(OH)_2_(*N*-nbdea)_4_((CH_3_)_3_CCOO)_6_(N_3_)_2_] and its core. Solvent molecules, disordered atoms and organic H atoms have been omitted for clarity. Dy blue; Fe green; O red; N blue; C black, H white.

Designing the ligand field environment can help to control the magnetic anisotropy of some of the later lanthanides [2–3], but this is less useful for the Dy^III^ ion. The unpredictable behaviour and the strong dependence of magnetic anisotropy and orientation of the easy axis of the magnetization of the Dy^III^ ion on very small changes in the ligand environment was predicted by ab-initio calculations [5]. But only recently, we have shown that such radical changes can also be seen experimentally. By using Mössbauer spectroscopy we have shown how minor changes in the electronegativity of the atoms in the ligand sphere and in the donor–acceptor nature of the substituents, and their position on the aromatic ring, can control the shape anisotropy of the Dy^III^ ions and, thus, their interaction with the iron centres [14–15]. Moreover, the reported compounds [14–15] – with an antiferromagnetic coupling in the central iron dimer – show a very intriguing effect: the collapse of the magnetic hyperfine splitting under the effect of the external magnetic field. This apparently paradoxical behaviour reveals how complex and case-sensitive the properties of the Fe–Ln clusters can be. One of the reasons for this effect may well be that the externally applied magnetic field can affect the ground state of Dy^III^ by lowering the energy of the system: This can result in a change of the direction of polarization, and thus the shape anisotropy of the Dy^III^ ion and its interaction with the iron centres can be controlled. This means that the Ln anisotropy can be influenced not only by altering crystal field, but also by an external source. But, surprisingly, this is not always the case. It seems that the structural aspect can prevail over the others. Here, we report how, contrary to reported Fe_2_Dy_2_ compounds [14–15], the application of an external magnetic field does not always affect the ground state of the Dy^III^ ion and its relaxation time. Two compounds [Fe_4_Ln_2_(μ_3_-OH)_2_(L)_4_((CH_3_)_3_CCOO)_6_(N_3_)_2_]·(solv) (Ln = Dy^III^, L = *N*-(*n*-butyl)diethanolamine, solv = 3(MeCN), **1**, see below in Figure 1) and Ln = Y^III^, L = *N*-methyldiethanolamine, solv = phenol, **2**) are magnetically and spectroscopically studied and their properties are compared.

## Results and Discussion

Compound **1** crystallizes in the triclinic space group *P*−1 with *Z* = 2; each Fe_4_Dy_2_ aggregate (Figure 1) occupies a general site with no crystallographically imposed symmetry. The central core of the aggregate possesses a {Fe^III^_4_Dy_2_(µ_3_-OH)_2_(µ_3_-OR)_2_} architecture, in which two of the Fe^III^ (Fe(1) and Fe(3)) and the two Dy^III^ ions, together with the two hydroxo ligands, are arranged in the well-known "butterfly" shape. The two Fe^III^ ions form the “wingtips” and the Dy^III^ ions define the “body” of the butterfly, with the two hydroxo ligands each bridging a FeDy_2_ triangle. The typical butterfly topology has a planar Fe_2_Dy_2_ unit, with the two (µ_3_-OH) bridges on opposite faces of the Fe_2_Dy_2_ plane. In contrast, in **1** the Fe_2_Dy_2_ butterfly is not planar; the two FeDy_2_ triangles show a dihedral angle of 43.8°, and the two hydroxo ligands are on the same face of the Fe_2_Dy_2_ unit.

Each of the four Fe^III^ ions in the aggregate is chelated by a fully-deprotonated (*n*-butyl)diethanolamine (nbdea) ligand. Of those chelating Fe(1) and Fe(3), one alkoxo arm bridges an Fe–Dy edge of the butterfly, while the other forms a (μ_3_-alkoxo) bridge between a Fe–Dy edge in the butterfly and a further Fe^III^ centre (Fe(2) or Fe(4)). The nbdea ligands chelating Fe(2) and Fe(4) then each form two (µ2-alkoxo) bridges, to a Dy atom and to an Fe atom in the butterfly. Peripheral ligation is provided by four µ-pivalato ligands in their common *syn,syn* bridging mode. Two further unidentate pivalates each coordinate to a dysprosium, with the non-coordinated carboxylate oxygen atom accepting a hydrogen bond from a (μ_3_-OH) ligand. The coordination is completed by two azido anions coordinated to the outer iron atoms (Fe(2) and Fe(4)). All four Fe ions are six-coordinate with distorted octahedral geometries, while the Dy^III^ ions are eight-coordinate with coordination polyhedra that may best be described as distorted bicapped trigonal prisms. The molecular structure of the Fe_4_Y_2_ aggregate in **2** is very similar to that of the Fe_4_Dy_2_ in **1**, and differs only in the replacement of the *n*-butyl groups in **1** by methyl groups and the replacement of Dy^III^ by Y^III^.

**Figure 2:**
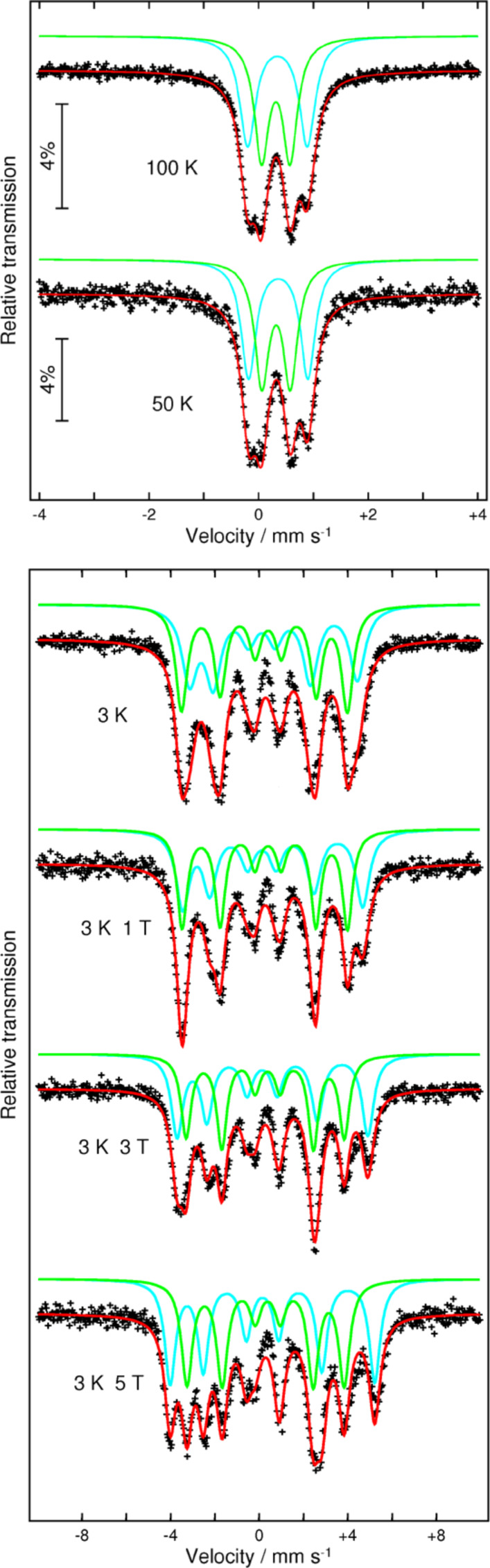
The **^57^**Fe Mössbauer spectra for [Fe_4_Dy_2_(OH)_2_(*N*-nbdea)_4_((CH_3_)_3_CCOO)_6_(N_3_)_2_] at 100 and 50 K (top); at 3 K in zero-applied magnetic field and at 3 K in applied magnetic fields of 1, 3 and 5 T (bottom). See Table 1 for the fitting parameters.

At temperatures higher than 20 K the Mössbauer spectra (MS) of **1** are paramagnetic (Figure 2) and show two symmetric quadrupole doublets with average isomer shifts, δ, of 0.42 and 0.46 mm/s and quadrupole splittings, Δ*E*_Q_, of 0.51 and 1.08 mm/s, respectively, (Table 1). This is typical for high spin, *S* = 5/2, Fe^III^ ions of iron-oxo proteins and other relevant model compounds [16–18]. The presence of two doublets with different quadrupole splittings (Δ*E*_Q_) indicates two Fe sites with different coordination spheres, in agreement with both the molecular structure and bond length distortion (BLD) calculations: Fe(1) and Fe(3) = 3.95 and 3.82, Fe(2) and Fe(4) = 3.30 and 3.19, respectively [19]. Consequently, we constrained the area ratio of doublets to 1:1. The low temperature (30 K) MS (not given here) for **1** is broad and shows a small onset of relaxation at an intermediate rate superimposed on the absorption peaks at the centre of the spectrum. At 3 K (Figure 2), a magnetic spectrum is obtained, indicating that the spin-relaxation time is slow with respect to the Mössbauer time scale. The spectrum has been fitted with two sextets with the parameters listed in Table 1. The best fits for the zero-field spectrum at 3 K were achieved through a free iteration method with two sextets with the effective hyperfine fields *B*_eff_ = 23.5 and 23.2 T, respectively, at the nuclei.

**Table 1:** Mössbauer data for [Fe_4_Dy_2_(OH)_2_(*N*-nbdea)_4_((CH_3_)_3_CCOO)_6_(N_3_)_2_] (**1**).

*T* [K]	Fe sites	δ^a^ [mm/s]	Δ*E*_Q_ or *ε*^b^ [mm/s]	Γ [mm/s]	θ [°]	φ [°]	*B*_eff_ [T]^c^

100	Fe_1,3_	0.45(1)	1.09(1)	0.35(3)	—	—	—
	Fe_2,4_	0.42(1)	0.52(2)	0.32(3)			

50	Fe_1,3_	0.47(2)	1.08(5)	0.32(1)	—	—	—
	Fe_2,4_	0.43(2)	0.52(4)	0.31(1)			

3	Fe_1,3_	0.48(1)	0.52(1)	0.83(1)	58	36	23.5(1)
	Fe_2,4_	0.43(2)	−0.17(1)	0.64(1)	52	70	23.2(1)

3 K, 1 T	Fe_1,3_	0.48^d^	0.49(1)	0.73(1)	51	37	25.2(5)
	Fe_2,4_	0.43^d^	−0.14(1)	0.55(1)	62	67	23.1(1)

3 K, 3 T	Fe_1,3_	0.48^d^	0.47(1)	0.63(1)	50	38	26.7(2)
	Fe_2,4_	0.43^d^	−0.11(1)	0.56(1)	63	64	22.1(1)

3 K, 5 T	Fe_1,3_	0.48^d^	0.45(1)	0.59(1)	49	39	28.6(1)
	Fe_2,4_	0.43^d^	−0.09(1)	0.60(2)	64	62	22.0(1)

^a^Relative to α-Fe at room temperature; ^b^For magnetically-split spectra the quadrupole shifts, ε = ½Δ*E*_Q_(3cos^2^φ − 1). φ - Euler angle between the internal hyperfine field, *B*_int_, and the principal axis (*V*_zz_) of the electrical field gradient. The quadrupole shifts are easy to observe from the magnetic spectra as a difference in the splitting of 1 and 2 and 5 and 6 iron(III) lines. δ - isomer shift, Δ*E*_Q_ - quadrupole splitting, θ - angle between *B*_eff_ and the direction of the γ-rays. The statistical errors are given in parentheses. The relative areas for the doublets and sextets have been constrained to a 1:1 ratio. ^c^*B*_eff_* = B*_int_* + B*_appl_; ^d^Fixed values.

When applying an external magnetic field one cannot see significant changes in the values of the hyperfine parameters. The spectra represent a superposition of two sextets with an area ratio 1:1. When the applied magnetic field is increased a behaviour that is typical for antiferromagnetically coupled molecular clusters can be seen [20–21]. The MS for **1** at 3 K, under the application of an external field (*B*_appl_ = 1, 3 and 5 T), are shown in Figure 2. Since the high-spin Fe^III^ ions are considered isotropic, it is expected that the local spins will align along (parallel or antiparallel) the direction of the applied field. Information about such an alignment can be derived from the relative intensity of the Δ*m* = 0 and Δ*m* = 1 lines of the sextet, which yield information about the angle θ between *B*_eff_ and the γ-rays. It is important to note that upon application of the external field the intensity of the *Δ*m = 0 lines does not change considerably (θ is almost constant) as it is to be expected for systems dominated by internal magnetic anisotropies. This indicates that even though the net moment of the cluster may be aligned along the direction of the applied magnetic field, the local magnetic moments tend to align approximately perpendicular to it. It appears that for **1** an applied field much larger than 5 T would be necessary to align the local magnetic moments completely along the direction of the field. An obvious change of the magnetization direction is suggested by the variation of the angle φ between the principal axis (*V_zz_*) of the electrical field gradient (EFG) and *B_int_*. This angle can be calculated from the magnetic spectra according to the formula ε = 1/2 Δ*E*_Q_(3 cos^2^φ −1), where ε are the quadrupole shift values determined from the magnetically ordered spectra and Δ*E*_Q_ are the quadrupole splitting values determined from the paramagnetic spectra. A clear variation of the φ values for both sextets is observed from 36° at 3 K (without a magnetic field) to 39° at 3 K (magnetic field 5 T) for Fe_1,3_ and from 70° at 3 K (without a magnetic field) to 62° at 3 K (magnetic field 5 T) for Fe_2,4_, respectively.

Another peculiarity observed from the magnetic spectra under an applied magnetic field is that one sextet is showing decreasing *B*_eff_ values regardless of the increased *B*_appl_, while the second one shows an increased value for *B*_eff_. This is a microscopic confirmation of the magnetic structure with Fe_1,3_ and Fe_2,4_ moments being antiferromagnetically coupled. The decrease and increase of the *B*_eff_ values at the nuclei with an increasing value of applied magnetic field is proof that the directions of the effective magnetic flux densities at the iron nuclei of iron have either the same or the opposite direction as the applied magnetic field. However, the difference in the change in the measured effective magnetic field for Fe_1,3_ and Fe_2,4_ sites is very different: 5.1 T and 1.2 T, respectively. The change of *B*_eff_ with *B*_appl_ for the Fe_1,3_ sites is close to 1.0, which is simply the applied field. This means that *B*_int_ (≈23.4 T) for these iron centers has a maximum (saturation) value. Contrary to Fe_1,3_, the change for Fe_2,4_ is very small. We anticipate an almost identical *B*_int_ for all sites and the above mentioned difference is due to a different reaction of the iron moments from the spin-coupled pairs to the applied field. It seems that the Fe_1,3_ moments tend to align themselves parallel to the applied field, but those of Fe_2,4_ have a spin-flop tendency into the plane perpendicular to the applied magnetic field. This could also be an explanation for the opposite evolution of the θ values (one is increasing and the other one is decreasing) with increasing *B*_appl_. Since the applied external magnetic field is perpendicular to the direction of γ-rays, the θ values should increase. The same conclusion can be made from the evolution of the Euler angles with *B*_appl_. While the angle φ for Fe_1,3_ is almost invariable (it changes from 36° to 39°), a more visible and opposite change from 70° to 62° can be seen for Fe_2,4_, together with a tendency to smaller angles, i.e., angles that deviate less from the direction perpendicular to *B*_appl_, or parallel to the γ-rays, respectively. Such unusual features can be attributed to the presence of an antisymmetric exchange in the diferric units. But such conclusions should be treated with caution, because of the unknown role of the very anisotropic Dy^III^ ions in this exchange.

**Figure 3:**
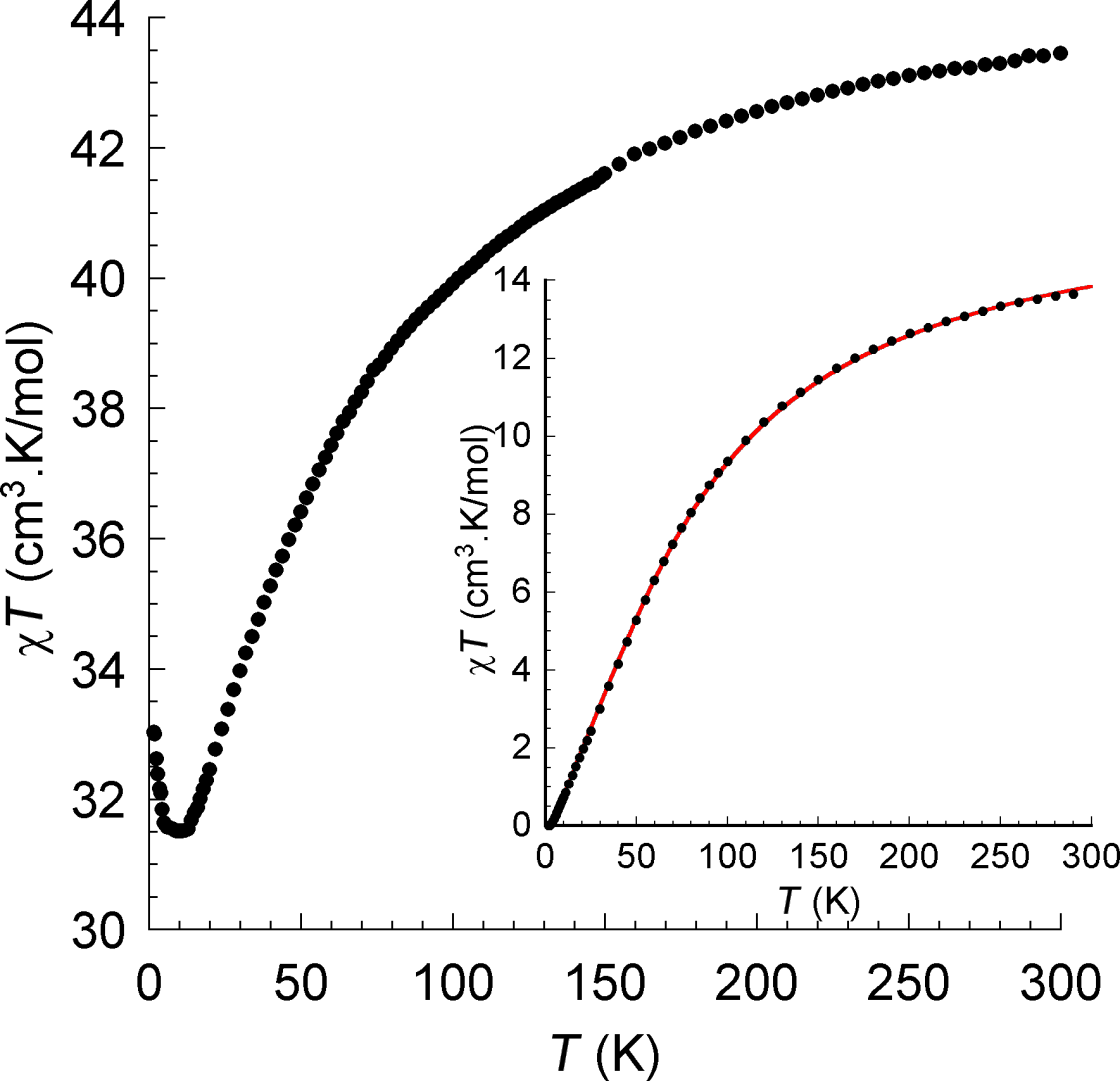
χ*T*-vs-*T* plots at 0.1 T for **1** and **2** (inset). The solid line is the best fit to the experimental data.

To shed some light on the interaction inside of these clusters, the direct-current (dc) magnetic susceptibility of **1** has been measured in an applied magnetic field of 0.1 T between 300 and 1.8 K (Figure 3). At room temperature, the χ_M_*T* value of 43.44 cm^3^·K·mol^−1^ is close to the expected value of 28.33 cm^3^·K·mol^−1^ for two uncoupled Dy^III^ ions (*S* = 5/2, *L* = 5, ^6^H_15/2_, *g* = 4/3) plus the value obtained for the iron fragments from the Y^III^ analogue. As shown in Figure 3, χ_M_*T* decreases with lowering temperatures in the range from 300 to 8 K and then increases sharply to reach a maximum of 33.00 cm^3^·K·mol^−1^ at 1.8 K, which may be because of an intramolecular ferromagnetic interaction. If the Fe–Fe (Fe(1)–Fe(2) and Fe(3)–Fe(4)) interaction is antiferromagnetic and the Fe–Dy exchange interaction is negligible, then the only ferromagnetic interaction is between the Dy ions of the central dimer. This is not unusual taking into consideration the relatively small Dy–Dy separation (3.87 Å) and angles of the Dy(1)–O(1)–Dy(2) and Dy(1)–O(2)–Dy(2) bridges (≈ 108°). To confirm the antiferromagnetism for the Fe(1)–Fe(2) and Fe(3)–Fe(4) units, the Y^III^ analogue **2** has been synthesized. At room temperature the value of χ_M_*T* is 13.5 cm^3^·K·mol^−1^ (Figure 3, inset), a value below the theoretical value for four uncoupled Fe^III^ ions (17.5 cm^3^·K·mol^−1^). This indicates a weak antiferromagnetic interaction between the spin centers. When the temperature is lowered, χ_M_*T* decreases and reaches zero at 1.8 K. This indicates clearly that the complex possesses an *S* = 0 ground state at low temperatures. The experimental data of **2** were fitted to the expression for the molar susceptibility derived from the Hamiltonian *H* = −2*J*·*S*_1_·*S*_2_. The best fit to the χ*T*-vs-*T* curve gave *g* = 1.94(1) and an exchange parameter *J* = −8.18(1) cm^−1^. Interestingly, the obtained *J* value for **2** is rather close to the value, approx. −8.3 cm^−1^, obtained from the Gorun and Lippard [22] empirical relationship between *J* in diiron(III) complexes with oxide, hydroxide and alkoxide bridges and the parameter *P* that corresponds to the half of the length of the shortest Fe–O–Fe-bridge in the complex. This is also supported by the value *J* ≈ −8.7 cm^−1^, which was determined by using another magnetostructural correlation originally developed for dimers that utilizes both the Fe–O distances and the Fe–O–Fe angles [23] and was later on improved and extended to polynuclear topologies [24–25]. In conclusion, all these experimental and theoretical data unambiguously prove that iron ions from the dinuclear units in compounds **1** and **2** are antiferromagnetically coupled.

**Figure 4:**
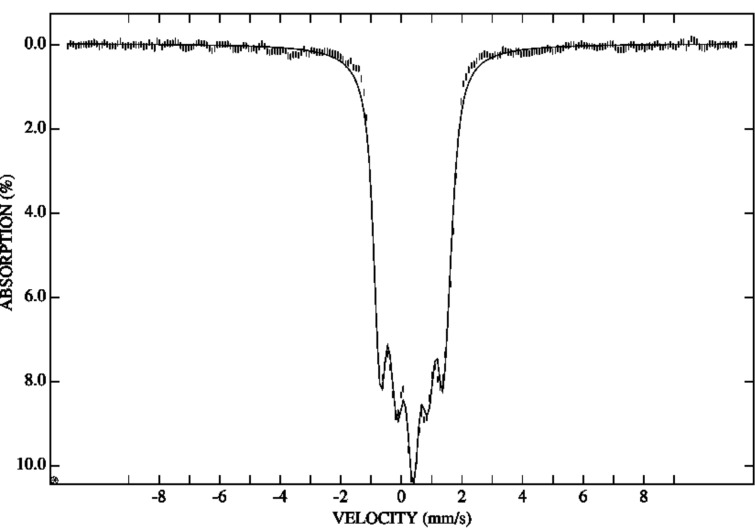
3 K Mössbauer spectrum of polycrystalline **2** recorded in a perpendicularly applied field of 5.0 T. The solid line is a spectral simulation for Δ*E*_Q_ = 0.80 mm/s, δ = 0.47 mm/s, and η = 0.95, assuming an isolated ground state with *S* = 0 for a dinuclear Fe_2_ cluster.

Additionally, the Mössbauer spectrum in an applied external magnetic field of 5 T has been measured for **2** (Figure 4). It confirms the diamagnetic ground state for the Fe_2_ units. The simulation parameters were the field at the nucleus *B*_eff_ = 5.0 T, Δ*E*_Q_ = 0.80 mm/s, δ = 0.47 mm/s, and the asymmetry parameter η = 0.95. The experimental data fit very well with *B*_eff_ = *B*_appl_, i.e., there is no noticeable contribution to the magnetic hyperfine interaction other than the applied field .

Mössbauer spectroscopy senses the internal hyperfine interactions near the nucleus of the studied isotope. There are four contributions that determine the internal hyperfine field *B*_int_ of an iron atom: *B*_fc_ (the Fermi contact interaction), *B*_ls_ (the orbital momentum of the 3d electrons at the nucleus), *B*_dd_ (the dipole field originating from the electronic shell) and *B*_latt_ (the lattice contribution). In both studied compounds the last three contributions can be neglected. The main contribution only results from *B*_fc_. But in an applied external magnetic field this contribution can also be ignored for compound **2**. The Mössbauer spectrum of compound **2** (Figure 4) shows that even under an applied field of 5 T, there is no orientation of the zero spin of the cluster and each antiferromagnetically coupled dimer is still relaxing fast on the Mössbauer time scale. As a result, a typical MS for an iron ion with zero ground state is obtained. However, this does not mean that this contribution can be neglected for compound **1**, too. This raises the question that if the Fe_2_ units in compound **1** are antiferromagnetically coupled as in compound **2**, why is the internal field felt by the iron nuclei so big to give a magnetic sextet? One contribution to the internal hyperfine field at the iron nuclei in compound **1** can result from the magnetic interaction with the anisotropic, magnetically aligned, Dy^III^ ions, which provide enough magnetic anisotropy to “block” the hyperfine field experienced by the iron nuclei. Another contribution can be a supertransferred field from Dy^III^ to Fe^III^ ions. At this time we are not confident which of the two contributions prevails.

But even without invoking a large supertransferred hyperfine field and without ignoring the contribution of the Fermi contact to the field, it is quite possible to qualitatively understand the Mössbauer spectra of both **1** and **2**. A determination of the zero-field splitting parameter for **1** would have been very useful, but it was not possible to determine. Depending on the sign of the zero field splitting parameter, *D*, in **1** at 3 K the iron(III) ions will be in a ground state that could be either |±5/2> or |±1/2>. If the relaxation between the positive and negative spin states becomes slow enough, the relaxation between the different *M*_S_ states may still be rather fast. In this case the effective hyperfine field that is observed is the Boltzmann average of the fields associated with the |±5/2>, |±3/2>, and |±1/2> states, an average that may well be close to 23 T. This can explain why the internal field observed at 3 K, is 23 T and not 55 T, a value expected for *S* = 5/2, which will be in agreement with values obtained for oxides (≈11 T per *S* = 1/2) [26]. Such an unusual slowdown of the relaxation may well be the result of an interaction of local Fe moments with the total magnetisation vector on the Dy^III^ dimer. For compound **2**, it is most probable that D is larger than zero and the ground state is |±1/2>. The reversal of the spin in this state, and hence of the hyperfine field, is very fast compared to the Mössbauer time scale and no hyperfine field is observed. Why is the relaxation fast? The relaxation in the |±1/2> state is particularly fast because it involves only a change of *M*_S_ of ±1 between the |+1/2> and the |−1/2> ground states.

A further peculiarity is the different evolution of the magnetic spectra with the external magnetic field. In contrast to previously reported studies about Fe_2_Dy_2_ clusters [6], in which the magnetic hyperfine splitting collapses under the application of an external magnetic field, in the case of **1** this applied field results in the total effective magnetic field decreasing for one sextet and increasing for the other. This confirms that the Fe_1_ and Fe_2_, and Fe_3_ and Fe_4_ pairs are antiferromagnetically coupled. No collapse of the magnetic structure is observed. An explanation may lie in the different communication between Dy and Fe ions. Contrary to previously reported Fe_2_Dy_2_ compounds, in which single Dy^III^ ions are in close proximity to either side of the Fe_2_ fragments, in compound **1** the Dy ions are on the same side and ferromagnetically cooperating with each other. This gives rise to a total ground spin state which cannot be affected by external applied magnetic field.

**Figure 5:**
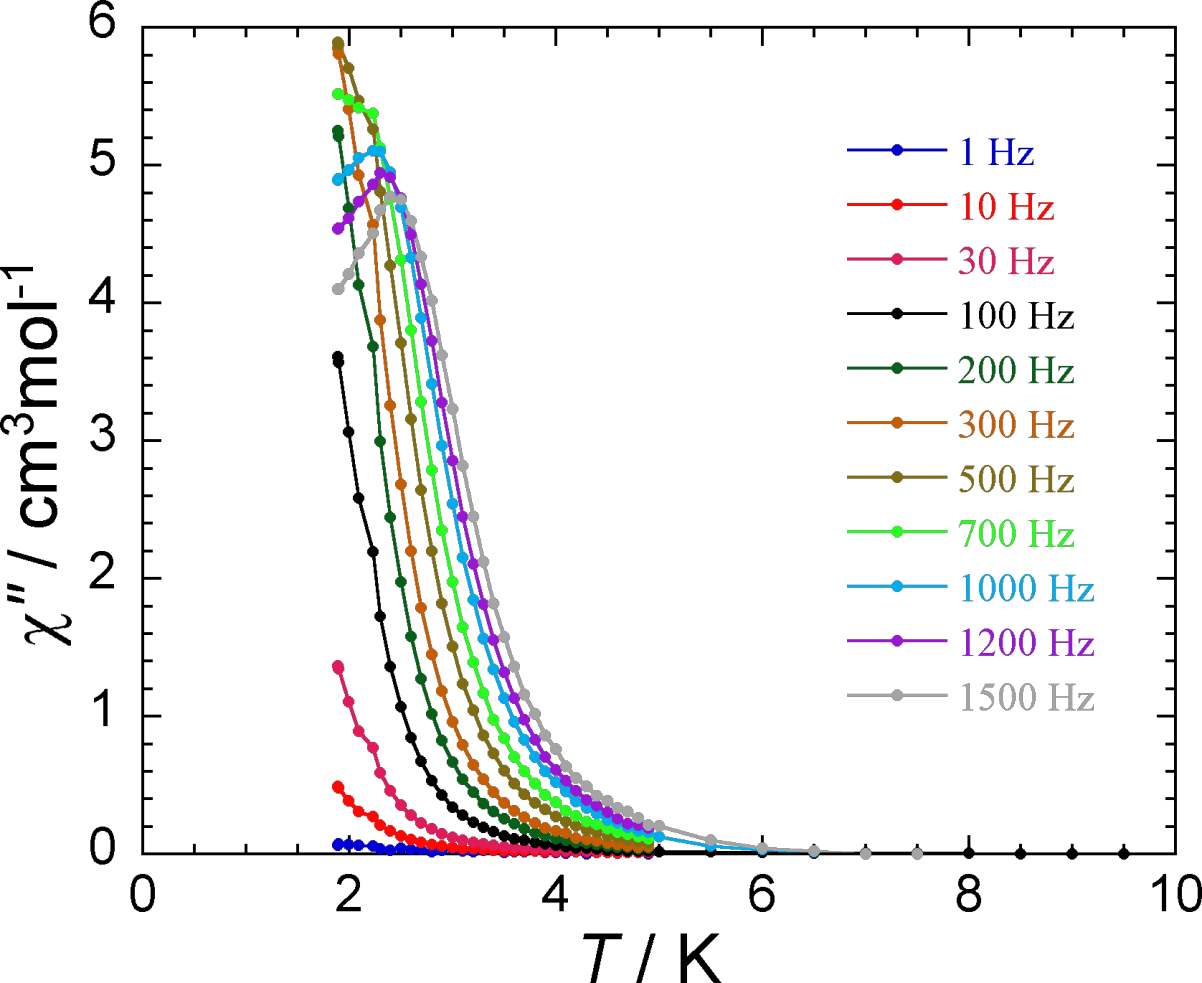
Plot of out-of-phase ac susceptibility signals vs temperature for **1** at the indicated oscillation frequencies.

It is worth mentioning that compound **1** is a single molecule magnet. It displays an out-of-phase response to the blocking temperature 2.5 K (at 1500 Hz), which is characteristic of a slow relaxation of the magnetization (Figure 5). The characteristic measuring time for Mössbauer spectroscopy is about 10^−7^ s, whereas that for ac magnetic susceptibility measurements is typically in the range between 10^0^ and 10^−4^ s (i.e., ac frequencies of 1–1500 Hz). Because of their different time windows, ^57^Fe Mössbauer spectroscopy and ac magnetic susceptibility measurements provide an apparently different view of the relaxation dynamic: If a slow relaxation can be seen in the magnetic data at very low temperatures (1.8–3.0 K), then an intermediate relaxation can already be seen in the Mössbauer spectra at 30 K. Therefore, an accurate comparative analysis of the data obtained from these two methods cannot be done.

## Conclusion

This communication once more shows how additional, case-sensitive information about the unpredictable lanthanide anisotropy can be gathered by using ^57^Fe Mössbauer spectroscopy. Due to the strong anisotropy of Dy ions, the magnetic susceptibility measurements provide only averaged information about the magnetic behaviour inside the molecules and one cannot distinguish all interactions inside the complex molecules. Mössbauer spectroscopy, on the contrary, provides us with microscopic information about the metal–metal communication and the relaxation dynamics on specific centres, in this case Fe nuclei in the presence of very anisotropic Ln centers. Having identified the nature of the interaction in compounds **1** and **2**, it will be of interest to explore the influence of incorporating other anisotropic lanthanides to shed light on the complex magnetism of lanthanide based SMMs.

## Experimental

Unless otherwise stated, all reagents were obtained from commercial sources and were used as received without further purification. All reactions were carried out under ambient conditions. Elemental analyses for C, H, and N were performed by using an Elementar Vario EL analyzer. IR spectra were measured on a PerkinElmer Spectrum One spectrometer as KBr disks.

**Preparation of [Fe_4_Dy_2_(OH)_2_(nbdea)_4_(O_2_CCMe_3_)_6_(N_3_)_2_]∙3MeCN (1):** A mixture of FeCl_2_ (0.127 g, 1.00 mmol), NaN_3_ (0.130 g, 2.00 mmol) and *N*-(*n*-butyl)diethanolamine (0.48 g, 3.00 mmol) in acetonitrile (15 mL) was stirred at room temperature for 20 min before adding 10 mL of dichloromethane. The obtained cloudy solution was stirred for another 10 min at 60 °C before the addition of Dy(NO_3_)_3_·6H_2_O (0.22 g, 0.50 mmol), pivalic acid (0.41 g, 4.00 mmol) and phenol (0.05 g, 0.50 mmol),. The mixture was further stirred until it became clear. The solution was filtered and left for slow evaporation. After one week red crystals of **1** were obtained. Yield 34.7% (based on Fe); Anal. calcd for C_66_H_133_Fe_4_Dy_2_N_13_O_22_ (**1**) (dried): C, 40.17; H, 6.59; N, 8.96; found: C, 40.98; H, 6.76; N, 6.73; IR (KBr) ν: 2948 (s); 2932 (s); 2877 (m); 2057 (s); 1603 (s); 1550 (s); 1476 (s); 1463 (w); 1407 (m); 1373 (m); 1352 (m); 1330 (w) 1284 (w); 1269 (w); 1222 (m); 1164 (w); 1142 (w); 1081 (m); 1054 (m); 1049 (m); 1019 (m); 998 (w); 936 (w); 904 (w); 885 (w); 877 (w); 814 (w); 788 (w); 754 (w); 695 (w); 625 (w); 606 (w); 585 (m); 513 (w); 470 (w); 426 cm^−1^ (w).

Complex **[Fe_4_Y_2_(OH)_2_(mdea)_4_(O_2_CCMe_3_)_6_(N_3_)_2_]^.^C_6_H_5_OH** (**2**) was obtained through the same procedure by using Y(NO_3_)_3_·6H_2_O in place of Dy(NO_3_)_3_·6H_2_O and mdea in place of nbdea. Yield 32.1% (based on Fe); Anal. calcd for C_56_H_106_Fe_4_N_10_O_23_Y_2_: C, 39.83; H, 6.32; N. 8.29; found: C, 39.93; H, 6.71; N, 8.12; IR (KBr) ν: 2961 (s); 2923 (s); 2872 (m); 2061 (s); 1601 (s); 1552 (s); 1481 (s); 1461 (w); 1407 (m); 1371 (m); 1354 (m); 1332 (w) 1286 (w); 1268 (w); 1226 (m); 1165 (w); 1142 (w); 1082 (m); 1058 (m); 1051 (m); 1024 (m); 998 (w); 935 (w); 902 (w); 886 (w); 876 (w); 814 (w); 786 (w); 753 (w); 698 (w); 626 (w); 607 (w); 587 (m); 548 (w); 512 (w); 472 (w); 425 cm^−1^ (w).

**Magnetic measurements:** The magnetic susceptibility measurements were carried out with a Quantum Design SQUID magnetometer MPMS-XL. This magnetometer works between 1.8 and 400 K for dc applied fields ranging from −7 to 7 T. Measurements were performed on polycrystalline samples. Alternating current susceptibility measurements have been measured with an oscillating ac field of 3 Oe and ac frequencies ranging from 1 to 1500 Hz. The magnetic data were corrected for the sample holder.

**Mössbauer spectroscopy:** The Mössbauer spectra were acquired using a conventional spectrometer in the constant-acceleration mode equipped with a ^57^Co source (3.7 GBq) in a rhodium matrix. Isomer shifts are given relative to α-Fe at room temperature. The sample was inserted inside an Oxford Instruments Mössbauer-Spectromag 4000 Cryostat, which has a split-pair superconducting magnet system for applied fields up to 5 T, with the field of the sample oriented perpendicular to the γ-ray direction. The sample temperature can be varied between 3.0 and 300 K.

**X-Ray crystallography:** Data were measured on Stoe IPDS II (**1**) or IPDS I (**2**) image plate diffractometers using graphite-monochromated Mo Kα radiation, and were corrected for polarisation and absorption. Structure solution (direct methods) and full-matrix least-squares refinement against *F*^2^ (all data) was carried out by using the SHELXTL software package [27]. All ordered non-H atoms were refined anisotropically; disordered atoms were refined with partial occupancy and geometrical restraints, either anisotropically with rigid-bond restraints or isotropically. Organic H atoms were placed in calculated positions; the positions of H atoms bonded to O were refined with O–H restrained to 0.92(4) Å. Crystal data for **1:** C_68_H_133_Dy_2_Fe_4_N_13_O_22_, 2033.27 g·mol^−1^, triclinic, *P*−1, *a* = 13.9237(6), *b* = 14.5196(7), *c* = 25.1289(10) Å, α = 82.856(3), β = 79.710(3), γ = 65.545(3)°, *Z* = 2, *V* = 4542.9(3) Å^3^, *T* = 150(2) K, ρ_calc_ = 1.486 g·cm^−3^, *F*(000) = 2088, μ(Mo Kα) = 2.315 mm^−1^; 62413 data, 21679 unique (*R*_int_ = 0.0342), 979 parameters, final *wR*_2_ = 0.1201, *S* = 1.018 (all data), *R*_1_ (17985 data with I > 2σ(I)) = 0.0449. **2**: C_56_H_106_Fe_4_N_10_O_23_Y_2_, 1688.72 g·mol^−1^, monoclinic, *P*2_1_/*c*, *a* = 19.1485(18), *b* = 16.4725(10), *c* = 24.797(2) Å, β = 101.162(11)°, *Z* = 4, *V* = 7673.5(11) Å^3^, *T* = 200(2) K, ρ_calc_ = 1.482 g·cm^−3^, *F*(000) = 3512, μ(Mo Kα) = 2.305 mm^−1^; 82403 data, 15075 unique (*R*_int_ = 0.0413), 917 parameters, final *wR*_2_ = 0.1073, *S* = 0.986 (all data), *R*_1_ (11528 data with I > 2σ(I)) = 0.0416. Crystallographic data (excluding structure factors) for the structures published in this paper have been deposited with the Cambridge Crystallographic Data Centre as supplementary publication nos. CCDC 957219 & 957220. Copies of the data can be obtained, free of charge, on application to CCDC, 12 Union Road, Cambridge CB2 1EZ, UK: e-mail: data_request@ccdc.cam.ac.uk, fax: +44 1223 336033 or http://www.ccdc.cam.ac.uk/cgi-bin/catreq.cgi.

## Supporting Information

File 1 CIF files for the crystal structures of **1** and **2**.
