# Supplementary material for: Spin relaxation in antiferromagnetic Fe–Fe dimers slowed down by anisotropic DyIII ions
Source: Beilstein J Nanotechnol. 2013 Nov 27;4:807–14. doi: 10.3762/bjnano.4.92 (PMC3869222; doi:10.3762/bjnano.4.92)
Supplement: File 1 — CIF files for the crystal structures of 1 and 2. [file Beilstein_J_Nanotechnol-04-807-s001.zip › L618_checkCIF.pdf]

checkCIF/PLATON report (basic structural check)

No syntax errors found. CIF dictionary
Please wait while processing .... Interpreting this report

Datablock: L618

Bond precision: C-C = 0.0080 A Wavelength=0.71073

Cell: a=13.9237(6) b=14.5196(7) c=25.1289(10)
alpha=82.856(3) beta=79.710(3) gamma=65.545(3)

Temperature: 150 K

	Calculated	Reported
Volume	4542.9(4)	4542.9(4)
Space group	P -1	P -1
Hall group	-P 1	-P 1
Moiety formula	C62 H124 Dy2 Fe4 N10 O22, 2(C2 H3 N), C2 N	C62 H124 Dy2 Fe4 N10 O22, 3(C2 H3 N)
Sum formula	C68 H130 Dy2 Fe4 N13 O22	C68 H133 Dy2 Fe4 N13 O22
Mr	2030.26	2033.27
Dx,g cm-3	1.484	1.486
Z	2	2
Mu (mm-1)	2.315	2.315
F000	2082.0	2088.0
F000'	2084.74	
h,k,lmax	18,19,33	18,19,33
Nref	21843	21679
Tmin,Tmax	0.481,0.615	0.472,0.610
Tmin'	0.472	

Correction method= MULTI-SCAN

Data completeness= 0.992 Theta(max)= 27.950

R(reflections)= 0.0449(17985) wR2(reflections)= 0.1201(21679)

S = 1.018 Npar= 979

The following ALERTS were generated. Each ALERT has the format
test-name_ALERT_alert-type_alert-level.
Click on the hyperlinks for more details of the test.

Alert level B

Crystal system given = triclinic
PLAT201_ALERT_2_B Isotropic non-H Atoms in Main Residue(s) 1
PLAT220_ALERT_2_B Large Non-Solvent C Ueq(max)/Ueq(min) ... 4.5 Ratio
PLAT413_ALERT_2_B Short Inter XH3 .. XHn H57D .. H74A .. 2.07 Ang.

Alert level C

PLAT041_ALERT_1_C Calc. and Reported SumFormula Strings Differ ? Check
PLAT068_ALERT_1_C Reported F000 Differs from Calcd (or Missing)... ? Check
PLAT202_ALERT_3_C Isotropic non-H Atoms in Anion/Solvent 3
PLAT213_ALERT_2_C Atom C47 has ADP max/min Ratio ..... 3.4 prola
PLAT222_ALERT_3_C Large Non-Solvent H Uiso(max)/Uiso(min) .. 6.1 Ratio
PLAT242_ALERT_2_C Check Low Ueq as Compared to Neighbors for C34
And 5 other PLAT242 Alerts
More ...
PLAT244_ALERT_4_C Low 'Solvent' Ueq as Compared to Neighbors of C71
PLAT244_ALERT_4_C Low 'Solvent' Ueq as Compared to Neighbors of C73

Alert level G

FORMU01_ALERT_2_G There is a discrepancy between the atom counts in the
_chemical_formula_sum and the formula from the _atom_site* data.
Atom count from _chemical_formula_sum:C68 H133 Dy2 Fe4 N13 O22
Atom count from the _atom_site data: C68 H130 Dy2 Fe4 N13 O22
CELLZ01_ALERT_1_G Difference between formula and atom_site contents detected.
CELLZ01_ALERT_1_G WARNING: H atoms missing from atom site list. Is this intentional?
From the CIF: _cell_formula_units_Z 2
From the CIF: _chemical_formula_sum C68 H133 Dy2 Fe4 N13 O22
TEST: Compare cell contents of formula and atom_site data

atom	Z*formula	cif sites	diff
C	136.00	136.00	0.00
H	266.00	260.00	6.00
Dy	4.00	4.00	0.00
Fe	8.00	8.00	0.00
N	26.00	26.00	0.00
O	44.00	44.00	0.00

PLAT002_ALERT_2_G Number of Distance or Angle Restraints on AtSite 23
PLAT005_ALERT_5_G No _iucr_refine_instructions_details in the CIF ? Do !
PLAT042_ALERT_1_G Calc. and Reported MoietyFormula Strings Differ ? Check
PLAT154_ALERT_1_G The su's on the Cell Angles are Equal .... 0.00300 Deg.
PLAT301_ALERT_3_G Note: Main Residue Disorder .... 3 %
PLAT302_ALERT_4_G Note: Anion/Solvent Disorder .... 33 %
PLAT793_ALERT_4_G The Model has Chirality at N1 (Verify) .... R
And 3 other PLAT793 Alerts
More ...
PLAT860_ALERT_3_G Note: Number of Least-Squares Restraints 50

0 **ALERT level A** = Most likely a serious problem - resolve or explain
3 **ALERT level B** = A potentially serious problem, consider carefully
13 **ALERT level C** = Check. Ensure it is not caused by an omission or oversight
14 **ALERT level G** = General information/check it is not something unexpected

6 ALERT type 1 CIF construction/syntax error, inconsistent or missing data
12 ALERT type 2 Indicator that the structure model may be wrong or deficient
4 ALERT type 3 Indicator that the structure quality may be low
7 ALERT type 4 Improvement, methodology, query or suggestion
1 ALERT type 5 Informative message, check

It is advisable to attempt to resolve as many as possible of the alerts in all categories. Often the minor alerts point to easily fixed oversights, errors and omissions in your CIF or refinement strategy, so attention to these fine details can be worthwhile. In order to resolve some of the more serious problems it may be necessary to carry out additional measurements or structure refinements. However, the purpose of your study may justify the reported deviations and the more serious of these should normally be commented upon in the discussion or experimental section of a paper or in the "special_details" fields of the CIF. checkCIF was carefully designed to identify outliers and unusual parameters, but every test has its limitations and alerts that are not important in a particular case may appear. Conversely, the absence of alerts does not guarantee there are no aspects of the results needing attention. It is up to the individual to critically assess their own results and, if necessary, seek expert advice.

Publication of your CIF in IUCr journals

A basic structural check has been run on your CIF. These basic checks will be run on all CIFs submitted for publication in IUCr journals (*Acta Crystallographica*, *Journal of Applied Crystallography*, *Journal of Synchrotron Radiation*); however, if you intend to submit to *Acta Crystallographica Section C* or *E*, you should make sure that full publication checks are run on the final version of your CIF prior to submission.

Publication of your CIF in other journals

Please refer to the *Notes for Authors* of the relevant journal for any special instructions relating to CIF submission.

PLATON version of 01/06/2013; check.def file version of 24/05/2013

Datablock L618 - ellipsoid plot

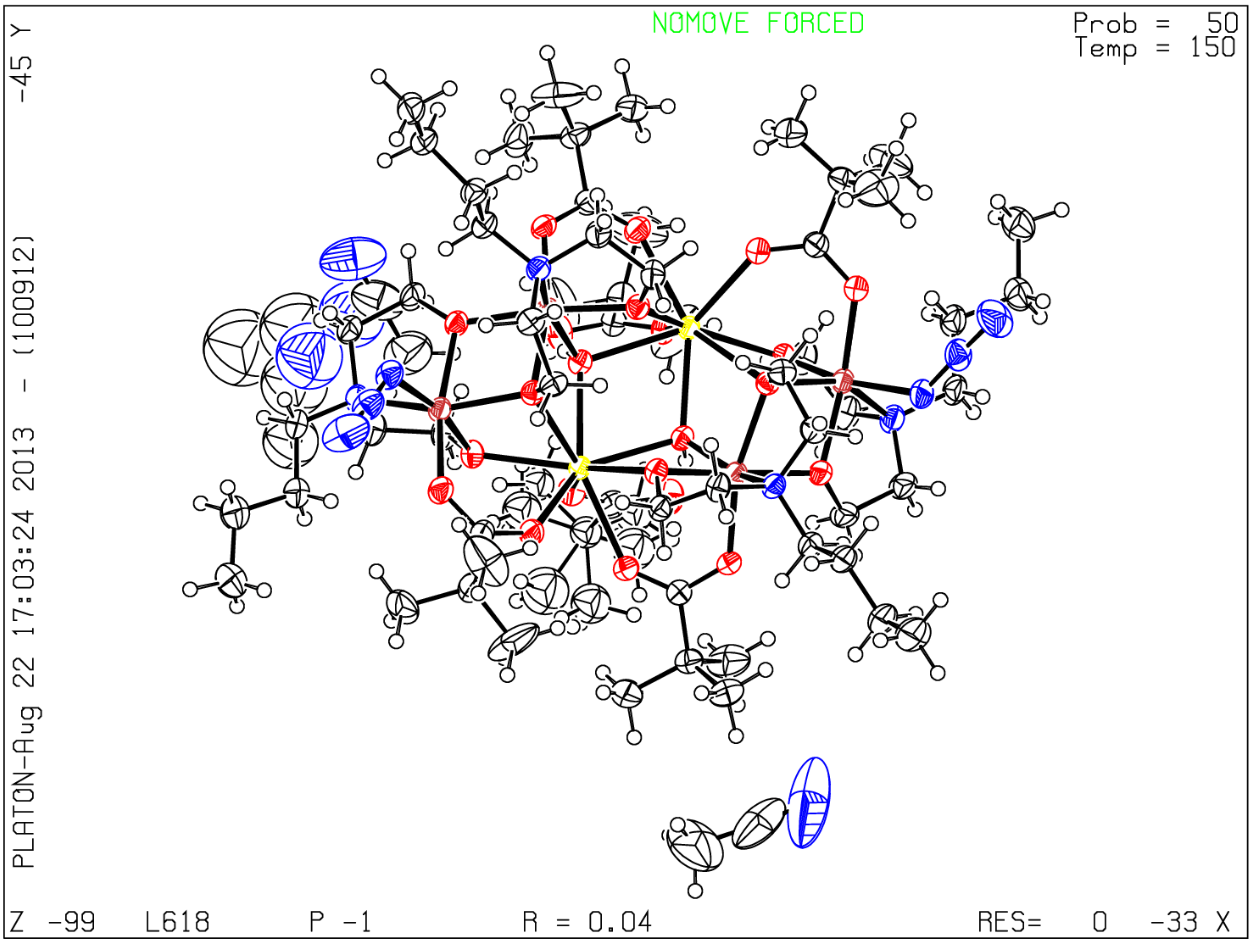

Download CIF editor (publCIF) from the IUCr
Download CIF editor (enCIFer) from the CCDC

Test a new CIF entry
