# Supplementary material for: Spin relaxation in antiferromagnetic Fe–Fe dimers slowed down by anisotropic DyIII ions
Source: Beilstein J Nanotechnol. 2013 Nov 27;4:807–14. doi: 10.3762/bjnano.4.92 (PMC3869222; doi:10.3762/bjnano.4.92)
Supplement: File 1 — CIF files for the crystal structures of 1 and 2. [file Beilstein_J_Nanotechnol-04-807-s001.zip › FKFe4Y2_checkCIF.pdf]

# checkCIF/PLATON report (basic structural check)

No syntax errors found.
Please wait while processing ....

CIF dictionary
Interpreting this report

# Datablock: FKFe4Y2

| | | |
|---|---|---|
| Bond precision: | C-C = 0.0065 A | Wavelength=0.71073 |
| Cell: | a=19.1485(18) b=16.4725(10) c=24.797(2) | |
| | alpha=90 beta=101.162(11) gamma=90 | |
| Temperature: | 200 K | |
| | Calculated | Reported |
| Volume | 7673.6(11) | 7673.5(11) |
| Space group | P 21/c | P 21/c |
| Hall group | -P 2ybc | -P 2ybc |
| Moiety formula | C50 H100 Fe4 N10 O22 Y2, C6 H6 O | C50 H100 Fe4 N10 O22 Y2, C6 H6 O |
| Sum formula | C56 H106 Fe4 N10 O23 Y2 | C56 H106 Fe4 N10 O23 Y2 |
| Mr | 1688.73 | 1688.72 |
| Dx,g cm-3 | 1.462 | 1.462 |
| Z | 4 | 4 |
| Mu (mm-1) | 2.305 | 2.305 |
| F000 | 3512.0 | 3512.0 |
| F000' | 3496.57 | |
| h,k,lmax | 23,20,30 | 23,20,30 |
| Nref | 15295 | 15075 |
| Tmin,Tmax | 0.530,0.616 | 0.557,0.671 |
| Tmin' | 0.519 | |

Correction method= MULTI-SCAN

Data completeness= 0.986 Theta(max)= 26.122

R(reflections)= 0.0416( 11528) wR2(reflections)= 0.1073( 15075)

S = 0.986 Npar= 917

The following ALERTS were generated. Each ALERT has the format
**test-name_ALERT_alert-type_alert-level**.
Click on the hyperlinks for more details of the test.

## Alert level B

Crystal system given = monoclinic
PLAT201_ALERT_2_B Isotropic non-H Atoms in Main Residue(s) ....... 6
PLAT220_ALERT_2_B Large Non-Solvent C Ueq(max)/Ueq(min) ... 4.2 Ratio
PLAT222_ALERT_3_B Large Non-Solvent H Uiso(max)/Uiso(min) .. 7.4 Ratio

## Alert level C

PLAT213_ALERT_2_C Atom C39 has ADP max/min Ratio ..... 3.3 prola
PLAT220_ALERT_2_C Large Non-Solvent N Ueq(max)/Ueq(min) ... 3.1 Ratio
PLAT241_ALERT_2_C Check High Ueq as Compared to Neighbors for C13
PLAT242_ALERT_2_C Check Low Ueq as Compared to Neighbors for N3
**And 6 other PLAT242 Alerts**
More ...
PLAT250_ALERT_2_C Large U3/U1 Ratio for Average U(i,j) Tensor .... 2.5
PLAT341_ALERT_3_C Low Bond Precision on C-C Bonds ............... 0.0065 Ang.
PLAT415_ALERT_2_C Short Inter D-H..H-X H44C .. H61B .. 2.13 Ang.
PLAT601_ALERT_2_C Structure Contains Solvent Accessible VOIDS of . 34 Ang3

## Alert level G

PLAT002_ALERT_2_G Number of Distance or Angle Restraints on AtSite 22
PLAT003_ALERT_2_G Number of Uiso or Uij Restrained non-H Atoms ... 23
PLAT005_ALERT_5_G No _iucr_refine_instructions_details in the CIF ? Do !
PLAT007_ALERT_5_G Note: Number of Unrefined Donor-H Atoms ........ 2
PLAT244_ALERT_4_G Low 'Solvent' Ueq as Compared to Neighbors of C61B
PLAT301_ALERT_3_G Note: Main Residue Disorder .................. 10 %
PLAT302_ALERT_4_G Note: Anion/Solvent Disorder .................. 100 %
PLAT720_ALERT_4_G Number of Unusual/Non-Standard Labels .......... 8
PLAT774_ALERT_1_G Suspect X-Y Bond in CIF: Y1 -- Y2 .. 3.86 Ang.
PLAT793_ALERT_4_G The Model has Chirality at N2 (Verify) .... R
**And 2 other PLAT793 Alerts**
More ...
PLAT811_ALERT_5_G No ADDSYM Analysis: Too Many Excluded Atoms .... ! Info
PLAT860_ALERT_3_G Note: Number of Least-Squares Restraints ....... 97

0 **ALERT level A** = Most likely a serious problem - resolve or explain
3 **ALERT level B** = A potentially serious problem, consider carefully
14 **ALERT level C** = Check. Ensure it is not caused by an omission or oversight
14 **ALERT level G** = General information/check it is not something unexpected

1 ALERT type 1 CIF construction/syntax error, inconsistent or missing data
17 ALERT type 2 Indicator that the structure model may be wrong or deficient
4 ALERT type 3 Indicator that the structure quality may be low
6 ALERT type 4 Improvement, methodology, query or suggestion
3 ALERT type 5 Informative message, check

It is advisable to attempt to resolve as many as possible of the alerts in all categories.

Often the minor alerts point to easily fixed oversights, errors and omissions in your CIF or refinement strategy, so attention to these fine details can be worthwhile. In order to resolve some of the more serious problems it may be necessary to carry out additional measurements or structure refinements. However, the purpose of your study may justify the reported deviations and the more serious of these should normally be commented upon in the discussion or experimental section of a paper or in the "special_details" fields of the CIF. checkCIF was carefully designed to identify outliers and unusual parameters, but every test has its limitations and alerts that are not important in a particular case may appear. Conversely, the absence of alerts does not guarantee there are no aspects of the results needing attention. It is up to the individual to critically assess their own results and, if necessary, seek expert advice.

### Publication of your CIF in IUCr journals

A basic structural check has been run on your CIF. These basic checks will be run on all CIFs submitted for publication in IUCr journals (*Acta Crystallographica*, *Journal of Applied Crystallography*, *Journal of Synchrotron Radiation*); however, if you intend to submit to *Acta Crystallographica Section C* or *E*, you should make sure that full publication checks are run on the final version of your CIF prior to submission.

### Publication of your CIF in other journals

Please refer to the *Notes for Authors* of the relevant journal for any special instructions relating to CIF submission.

---

**PLATON version of 01/06/2013; check.def file version of 24/05/2013**

# **Datablock FKFe4Y2** - ellipsoid plot

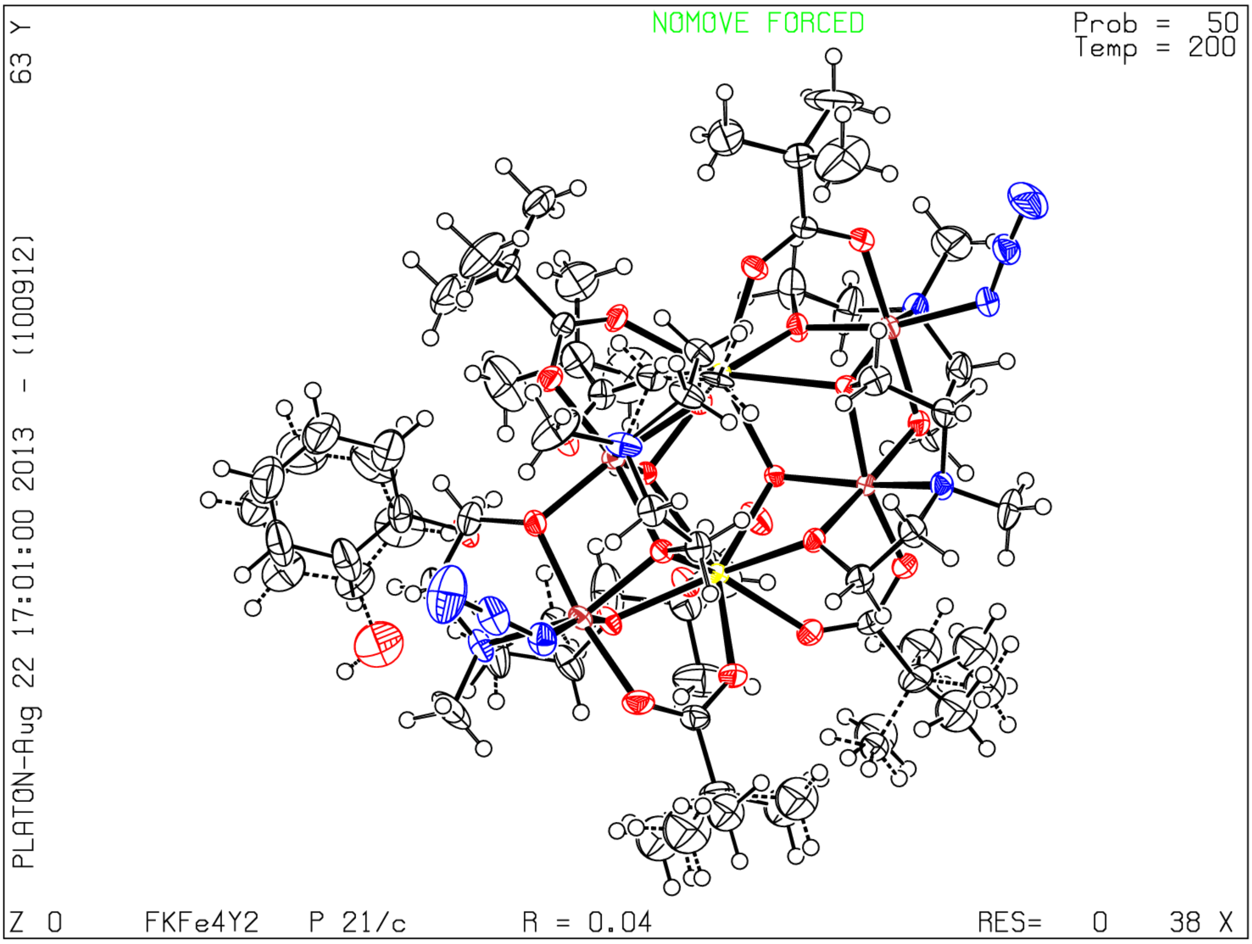


---

Download CIF editor (publCIF) from the IUCr
Download CIF editor (enCIFer) from the CCDC
Test a new CIF entry
